# A38 OVER-THE-SCOPE AND THROUGH-THE-SCOPE SUTURING OUTCOMES FOR ENDOSCOPIC LEAK AND FISTULA MANAGEMENT: A SYSTEMATIC REVIEW

**DOI:** 10.1093/jcag/gwae059.038

**Published:** 2025-02-10

**Authors:** D Flegg, C Thilakanathan, H Kim, D Motomura, S Gan, E Lam, R Trasolini, N Shahidi

**Affiliations:** Internal Medicine, The University of British Columbia Faculty of Medicine, Vancouver, BC, Canada; Internal Medicine, The University of British Columbia Faculty of Medicine, Vancouver, BC, Canada; Internal Medicine, The University of British Columbia Faculty of Medicine, Vancouver, BC, Canada; StPaul’s Hospital, Division of Gastroenterology, Vancouver, BC, Canada; Vancouver General Hospital, Division of Gastroenterology, Vancouver, BC, Canada; StPaul’s Hospital, Division of Gastroenterology, Vancouver, BC, Canada; Vancouver General Hospital, Division of Gastroenterology, Vancouver, BC, Canada; StPaul’s Hospital, Division of Gastroenterology, Vancouver, BC, Canada

## Abstract

**Background:**

Leaks and fistulae are well-recognized sequelae arising from gastrointestinal surgeries, inflammatory bowel disease, malignancy, and traumatic injury; reflecting significant morbidity, mortality and resource utilization. Endoscopic management has gained traction as a minimally invasive alternative to surgery including both over-the-scope suturing (OTSS) and through-the-scope suturing (TTSS).

**Aims:**

We aimed to systematically evaluate available evidence for OTSS and TTSS outcomes for leak and fistula management.

**Methods:**

From inception to July 1^st^, 2024, MEDLINE, EMBASE and Cochrane Library were searched for relevant citations. Full-text citations were included if they evaluated both technical success (endoscopic closure of leak or fistula) and clinical success (technical success with avoidance of further intervention) for leak or fistulae management with OTSS or TTSS. In this preliminary analysis, outcomes were reported as ranges.

**Results:**

A total of 11 studies including 185 defects were included for analysis. For fistulae, technical success for OTSS and TTSS ranged from 83-100% and 82-100%, respectively. Clinical success for OTSS and TTSS ranged from 0-80% and 55-100% respectively. For leaks, technical success for OTSS and TTSS ranged from 90-100% and 0-57%, respectively. Clinical success for OTSS and TTTS ranged from 25-100% and 29%, respectively.

**Conclusions:**

OTSS and TTSS are new interventions for leak and fistulae management with varying performance outcomes. Further research is required to delineate optimal candidates for OTSS/TTSS closure and treatment algorithms after technical/clinical failure.

**Funding Agencies:**

None

